# Repair of old total perineal rupture: a case series

**DOI:** 10.1093/jscr/rjac628

**Published:** 2023-01-17

**Authors:** Suskhan Djusad, Anggrainy Dwifitriana Kouwagam

**Affiliations:** Division Urogynecology and Reconstruction, Department of Obstetrics and Gynecology, Dr. Cipto Mangunkusumo National Central Public Hospital, Jakarta, Indonesia; Division Urogynecology and Reconstruction, Department of Obstetrics and Gynecology, Dr. Cipto Mangunkusumo National Central Public Hospital, Jakarta, Indonesia

## Abstract

Obstetric anal sphincter injuries are complications occurred during vaginal delivery. An inadequate of this condition may lead to the total perineal rupture. One of the symptoms is anal incontinence. This condition may interfere with the patient’s quality of life. This study aims to present symptoms, risk factors and surgical management in old total perineal rupture cases. We presented seven cases of old total perineal rupture, from March 2021 until March 2022. All the patients had a chief complaint of anal incontinence. All patients had previous primary suturing at the time of delivery. From the sphincter US examination, all the patients had a defect on the internal and external ani sphincter. The overlapping sphincteroplasty and perineorrhaphy technique was performed in all patients. Identification of perineal lacerations and their immediate treatment are very important to prevent the occurrence of old total perineal rupture. Management with the overlapping sphincteroplasty and perineorrhaphy is an effective technique to correct this condition.

## INTRODUCTION

A perineal laceration can occur at the time of delivery. The severity of the tear depends on the extension of the laceration to the anal sphincter. The injuries to the anal sphincter occur in 1–9% of all cases of vaginal delivery. Of these incidence rates, 0.03–0.2% were fourth-degree cases of vaginal injuries. The injuries to the perineum can be diagnosed at the time of delivery so that treatment can be done immediately. However, in some areas with incomplete health facilities, perineal laceration is not treated adequately and causes a total perineal defect. In advanced conditions, perineal lacerations can cause anal incontinence. Anal incontinence is a condition in which the patient is unable to control flatus or stool, thus interfering with the quality of life [1].

In this case series, we presented seven cases of old total perineal rupture. The aim of this study is to present the risk factors that play a role in total perineum rupture and our surgical technique to repair the rupture.

## CASE SERIES

### Case 1

A woman, 27 years old, P1A0 came with chief complaints of not being able to hold flatus and defecation. The patient had spontaneous delivery by midwife with a birth weight of 3400 g. At the time of delivery, the midwife did not do abdominal pressure. After that, the midwife did the suturing. Seven days after delivery, the patient came for control and said that the sutures were open. The patient was resuturing with the surgeon. After that, the patient was referred to the obstetrician, the doctor said that all the suture was removed and would be re-sutured 3-month later. At that time, the patient had felt the feces coming out of the vagina and could not hold defecation. The patient was then referred to an advanced health facility for fistula surgery. After the operation, there was no stool coming out from the vagina but she was still unable to hold defecation. Wexner score was 13. On US examination, there were external anal sphincter (EAS) and internal anal sphincter (IAS) defects at 10–2 o’clock. Three months after surgery, the wexner score was zero.

### Case 2

A woman, 30-year old, P1A0 with chief complaints of stool coming out from the vagina after 2-day postpartum. The patient had spontaneous delivery by midwife with a birth weight of 2700 g. An episiotomy and abdominal pressure were performed. The complaints got worse until the patient was unable to hold solid and soft stools. There was no history of the previous repair. Wexner score was 16. On ultrasound found, EAS and IAS defects at 9–3 o’clock. Three months after surgery, the wexner score was zero. From the US test, there was no defect on external and internal anal sphincter.

### Case 3

A woman, 27 years old, P1A0. Post spontaneous delivery by a midwife with a birth weight of 3200 g. The patient underwent an episiotomy and abdominal pressure. Two weeks postpartum, the suture was open. However, the patient did not continue the treatment. Eight months postpartum, the patient complained of uncontrolled flatus and solid or liquid stool. Wexner score was 15. On the US sphincter, there were EAS and IAS from 10 to 2 o’clock. Three months after surgery, the wexner score was zero and there was no defect on sphincter ani.

### Case 4

A 41-year-old woman with P2A0 with chief complaints of not being able to hold liquid stools and flatus since 11 years ago. The patient had spontaneous delivery by the midwife. At the time of delivery, the patient was not performing episiotomy and abdominal pressure. The birth weight was 3800 g. Five days after postpartum, the patient felt that feces were coming from the vagina. Three months later, the patient came to obstetricians to change the intrauterine Device (IUD), the doctor said the IUD could not be removed because there was a myoma in the cervical canal. The patient was then referred and told that she also had an old total perineal rupture. Currently, the patient felt flatus and liquid stools always come out of the vagina, sometimes solid stools too. Wexner score was 18. On the US sphincter, there was a defect in the EAS at 9–1 o’clock and the IAS at 10–1 o’clock. Three months after surgery, the wexner score was decrease until zero score. From the sphincter US test, there was no defect on external and internal anal sphincter.

### Case 5

A woman, 33 years old, P3A2 with chief complaints of feces coming out from vagina. The patient had spontaneous delivery by the midwife with a birth weight of 3800 g. The midwife said, there was a laceration from the perineum to the anus, the patient was referred to the hospital but there was no obstetrician so the midwife had sutured it there. Three days postpartum, the patient was controlled by to doctor and the doctor said that the suture loosen. Then, she was referred to a tertiary hospital. Eight days after postpartum, the patient felt feces coming out of the anus, but there were also some coming out from the vagina. She felt flatus from her anus. Wexner score was 14. On the US sphincter, there was an external and internal anal sphincter defect at 9–3 o’clock. From the Sphincter US examination after three months surgery, there was no defect on external and internal anal sphincter. The wexner score was zero.

### Case 6

A woman, 29 years old, P1A0, came with a chief complaint of not being able to control her flatus and stools. The patients had spontaneous delivery by the midwife. She was performed abdominal pressure without episiotomy. The midwife said she had a laceration to the anus and the midwife there sutured it. Five days postpartum, the patient felt the suture open and the midwife re-sutured. Three days later, the patient felt the stitches open again so she was referred to a secondary hospital. The patient was told that she had a total perineal rupture. Currently, the patient has difficulty controlling flatus, liquid stools, and sometimes solid stools. Wexner score was 13. On the US sphincter, there was a defect in the external and internal anal sphincter at 10–2 o’clock. Three months follow up after surgery, The wexner score was zero and there was no defect on sphincter US examination.

### Case 7

A woman, 29 years old, P2A0 with chief complaints of not being able to control her flatus and stools. The patient was post-delivery by the midwife with a birth weight of 4100 g. No episiotomy and abdominal pressure were performed. The patient was sutured by the midwife and the degree of laceration was not stated. After 10-day postpartum, the patient felt the sutures open and there were feces in the vagina. The patient was then referred to the secondary hospital. At this time the patient felt difficulty controlling flatus and liquid stools, sometimes the solid stools too. Wexner score was 12. On the US sphincter, there was a defect in the external ani and internal ani sphincter at 10–2 o’clock. Three months after surgery, we did the repeated US test. The result was no defect on sphincter ani eksterna and interna. The wexner score was decrease to one score.

**Figure 1:**
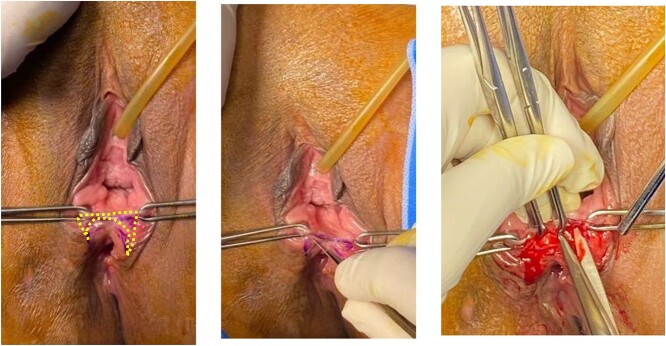
Butterfly-shape incision was made along the junction of posterior vaginal mucosa and rectal mucosa. Sharp dissection was done to separate anterior wall of rectal mucosa from posterior vaginal wall creating a good rectovaginal space.

**Figure 2:**
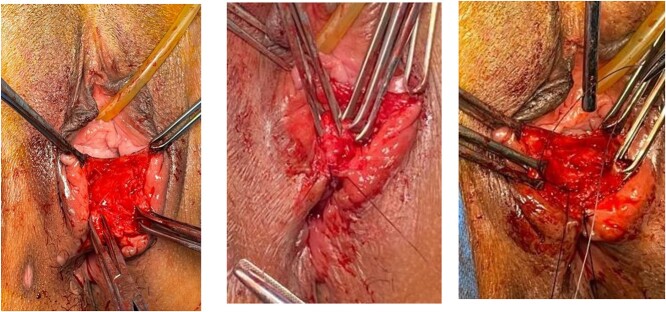
Identified anal mucosa, EAS and IAS. Anal mucosa was stitched interruptedly with an intraluminal knot. IAS was stitched with horizontal mattress.

**Figure 3:**
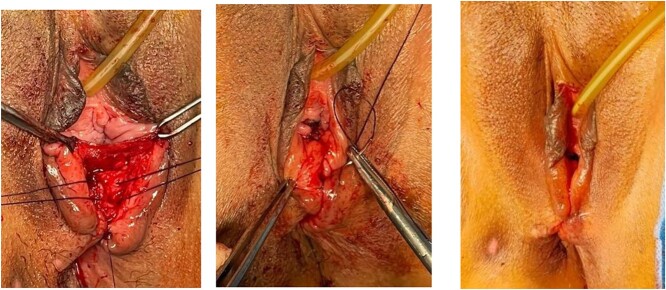
EAS was stitched overlapping technique. Perineal muscle, vaginal mucosa and perineal skin were stitched. Continued with perineorrhaphy using PGA No. 2.0.

In this case series, all patients were repaired at Cipto Mangunkusumo Hospital Jakarta, at least 3 months after the previous repair. The detailed surgical procedures were depicted in Figs 1–3 as shown below.

## DISCUSSION

Vaginal lacerations often cause injury to the perineal body, anal sphincter complex and anorectal mucosa. The risk factors of perineal injury are Asian ethnicity, nullipara, birth weight of >4 kg, shoulder dystocia, occipito-posterior position, the prolonged second stage of labor and use of instruments during delivery. As a result of the injury, the perineal body is no longer formed and is separated from the anal sphincter [1, 2].

From several prospective studies, it was found that patients with grades 3B to grade 4 had a higher risk of anal incontinence. This is due to the IAS’s role in the maintenance of continence. The identification of IAS injury may be difficult, therefore a detailed examination should be carried out prior to suturing, including a digital rectal examination. Management anal incontinence can also occur due to damage to the pudendal nerve during prolonged vaginal delivery [3].

The identification of the degree of laceration and primary repair are steps that are efficient, cost-effective and improve the quality of life. However, sometimes the health workers who assist in childbirth cannot identify the degree of injury so the treatment becomes inadequate. The secondary repair can be performed, especially on patients with previous failed repairs, or by using the Wexner score. The Wexner score is an instrument used to assess the efficacy of surgical therapy for anal incontinence. The scoring assesses the frequency and type of incontinence such as solid, liquid, gas, wear pad and lifestyle alteration. The maximum score is 20. A score of >12 indicates a significant abnormality that requires surgical treatment [4, 5].

Before surgery, endoanal ultrasound may be performed to determine the type of anal sphincter injury and assess the injury of the pudendal nerve [4]. The aim of surgery is to restore the internal and EAS structures. A good reconstruction can result in the lengthening of the anal canal and return of function. Epidural anesthesia may be an option for reconstructive surgery. In addition, the anesthesia performed can also relax the anal sphincter so that anal sphincter fusion can occur without tension [6].

The use of delayed absorbable materials such as polyglactin 910 and polyglycolic acid can be used when suturing. This material does not cause much pain or the need for analgesia or the dehiscence [6].

To repair a perineal laceration, several types of surgical techniques can be performed. One of them is the repaired in layers technique as seen in our case. The IAS can be sutured separately. In this layer, a very good reapproximation, the right strength, and integrity of the repair are required. This is the because of important role of IAS in maintaining continence. The EAS appearance will appear darker than IAS. EAS repair can be done by end-to-end or overlapping plication of the disrupted muscle. The EAS capsule can be sutured with an interrupted or figure of eight technique. After that, the perineal muscles can be sutured to support the anal sphincter.

The end-to-end technique sometimes does not always end with a good outcome. Therefore, the obstetricians use overlapping repair to correct the defect and fecal incontinence with a better outcome. On the other side, the study by Farrel *et al*. showed no difference in long-term outcomes from the end-to-end and overlapping techniques [7]. For suturing vaginal tissue, perineal muscles and skin, the continuous non-locking suturing technique can be used. A rectovaginal examination should be performed after the repair to ensure the repair has been carried out properly. Postoperative improvement of symptoms can occur in 3–6 months postoperatively [4].

## CONCLUSION

Early identification of the severity of perineal laceration is important. The primary repair management should be done when the laceration is recognized. Surgical technique by overlapping sphincteroplasty and perineorrhaphy can be performed to correct the old total perineal rupture. The surgery aims to reconnect the external and internal anal sphincter so that the anal continence function returns to normal.

## CONFLICT OF INTEREST STATEMENT

None declared.

## FUNDING

None.
